# Quality by Design-Guided Systematic Development and Optimization of Mucoadhesive Buccal Films

**DOI:** 10.3390/pharmaceutics15102375

**Published:** 2023-09-23

**Authors:** Alharith A. A. Hassan, Katalin Kristó, Yousif H.-E. Y. Ibrahim, Géza Regdon, Tamás Sovány

**Affiliations:** 1 Institute of Pharmaceutical Technology and Regulatory Affairs, University of Szeged, Eötvös u. 6., H-6720 Szeged, Hungary; alharith.hassan@szte.hu (A.A.A.H.);; 2 Department of Pharmaceutics, Faculty of Pharmacy, University of Khartoum, Khartoum P.O. Box 321, Sudan; 3 Pharmaceutics Department, Omdurman Islamic University, Omdurman P.O. Box 382, Sudan

**Keywords:** critical quality attributes, design space, enalapril maleate, lidocaine, mucoadhesive buccal film, quality by design, risk assessment

## Abstract

Mucoadhesive buccal films have found increased popularity in pharmaceutical drug delivery due to the several advantages that they possess. The present study strives to develop and optimize chitosan-based mucoadhesive buccal films by relying on quality-by-design (QbD) principles. Previous knowledge and experience were employed to firstly identify the critical quality attributes (CQAs), followed by a thorough risk assessment, which led to the selection of seven critical material attributes and process parameters, namely, the polymer grade and concentration, the plasticizer type and concentration, the citric acid (CA) concentration, the amount of the casted solution, and the drying condition. Their effects on the breaking hardness and mucoadhesivity, selected as CQAs, were investigated in three steps by three designs of the experiment (DoE). The medium molecular weight of chitosan (CH) was the preferred choice in the optimized formulation, and its concentration was the most important factor affecting the CQAs, thickness, and moisture content of the films. It was found that 0.364 g/cm^2^ was the suitable amount of the casting solution, and its optimum drying conditions were presented in the form of a design space. Glycerol (Gly) was the best choice as a plasticizer, and a design space representing several combinations of CH and CA concentrations that produce films with the required quality was constructed at a fixed concentration of 35% Gly. A formula from this design space was selected and employed to load with two model drugs to test its drug-carrying properties for drugs with different physicochemical characteristics. Uniform drug distribution with an immediate release profile was achieved in both drugs, although one of the CQAs was outside of the specifications in the case of lidocaine-containing film. To summarize, the obtention of the optimum mucoadhesive buccal film based on CH was efficiently facilitated by the rational application of QbD principles and the DoE approach.

## 1. Introduction

Buccal mucosa constitutes an attractive target for small molecular drugs and macromolecular and biological ingredient delivery intended for local or systemic administration [1,2,3,4,5,6,7,8,9]. Self-administration, reduced sensitivity, absence of pain, easy access, application, and removal increase patients’ compliance, so it could also be used to deliver currently parenterally administered active pharmaceutical ingredients (APIs). The buccal route shows superiority over the oral one in terms of a faster onset of action and improved bioavailability of APIs due to the relatively high blood flow, reduced enzymatic activity, bypassed first-pass metabolism, and lack of acidic degradation or food interactions [1,3,4,5,6,7,9,10,11,12,13]. Various dosage forms have been developed, including tablets, discs, mucoadhesive films, gels, ointments, creams, pastes, and sprays, to exploit the potentiality of this noninvasive delivery route for a variety of medicines [4,5,6,8,9,11,14,15,16]. However, there are several formulation challenges associated with this route that frequently disrupt the achievement of the required drug concentration locally or systemically. The salivary renewal cycle and masticatory effect during eating and drinking are among the major challenges that are most likely to reduce the drug contact time and significantly alter the drug distribution kinetics. Mucoadhesive films appear to be superior over others in mitigating these shortcomings and improving performance by ensuring intimate contact between the API and the buccal mucosal tissue for the required period, thus enabling a high drug concentration and enhancing permeation through the membrane to access the submucosal tissue and/or systemic circulation, in addition to constituting an effective barrier against mucosal enzymes, salivary secretions, electrolytes, and pH changes [4,5,9,13,16]. Moreover, buccal films appear to be more favorable compared to sublingual administration from the patient’s perspective, as they are less likely to interfere with usual activities, such as speaking, drinking, and mastication. Furthermore, they are thin and offer more flexibility and comfort compared to tablets [4,5,6,10,11].

Mucoadhesive films have been prepared by a variety of methods, but the most commonly used, especially at a small-scale research level, is the solvent casting (solvent evaporation) method. The popularity of this method is justified by the ease of its preparation and handling, flexibility, and low production costs [1,3,4,5,6,12,14,16,17].

The solvent casting method comprises four main steps: (a) dissolving or dispersing and mixing a film-forming polymer and other excipients in a volatile solvent, (b) casting the solution onto a plate, (c) drying the solution, (d) and peeling the dried films from the plate. Among these, drying is the most time-consuming step, especially with aqueous dispersions. Based on a thorough literature review, the effects of the drying conditions on the properties of the formulated films have not yet been comprehensively studied. Different and variable drying conditions have been reported, ranging from uncontrolled temperature and humidity under ambient conditions to higher controlled temperatures in an incubator or an oven. Consequently, varied durations of the drying time were reported and mostly ranged between 24 h and 72 h [1,2,3,4,5,10,12,16,16,18]. This wide variability is not only due to the variations in the used temperatures but also due to other formulation parameters. For instance, F. Kianfar et al. reported a significant increase in the drying time (almost double) upon the addition of polyethylene glycol (PEG) as a plasticizer in the formulation justified by the reduced evaporation rate of water due to the retention effect of the plasticizer [18]. Also, using different types and grades of film-forming polymers affect the drying rate, reflecting their interactions with the applied solvent [18].

Another attempt was carried out by Kazsoki et al., who investigated the transition of gel into a film while drying in the open air, which is a slow process. The structural changes and transitions that occur at the molecular level in the polymer and API were tracked using real-time ortho-positronium annihilation to determine the endpoint of the process with films containing the equilibrium moisture content [19].

Visser and co-workers reported the development and optimization of orodispersible films based on the quality-by-design (QbD) approach. The effects of three factors, including the drying temperature, were investigated. The relatively short drying time at 30 °C reported by the authors suggests the application of an extra tool to accelerate drying, such as the utilization of a vacuum or forced air stream, which was not stated. Moreover, the drying time factor, which considerably influences the ease of preparation, was not included in the design of the experiment (DoE) [20].

Very recently, Calvo et al. investigated the effects of five factors on the quality of mucoadhesive films designed for local delivery on another mucosal surface (vagina). They introduced temperature as one of the process parameters and dried the films casted on Petri dishes in an oven at two different temperatures. It was shown that the optimized film was obtained at a lower temperature (30 °C). Again, it was not reported whether this drying was aided by an airflow created by, e.g., a fan or not, as this factor might affect the film properties and could highly influence the drying time, which was not included as a response in this work. One of the studied responses was the film thickness, aiming to obtain films as thin as possible. However, one of the factors that could affect this response, as well as other film criteria, was not involved, which is the amount of the casted solution [21].

Film-forming polymer is the main component and represents the backbone of the mucoadhesive systems. Other ingredients include plasticizers, penetration (or permeation) enhancers, and enzyme inhibitors [5,6,8,9,13,22]. Among these, the plasticizer is the most commonly used and crucial excipient to modify the mechanical properties of the films [5,17,21].

QbD principles have increasingly been employed in a variety of research and industrial activities, with more importance in pharmaceutical product developments [23,24]. This systematic approach is highly efficient in developing pharmaceutical products because it is based on previous comprehensive knowledge and a thorough assessment of all risk factors that could affect the quality of the product. It generally begins with a clear determination of the target in terms of the quality target product profile (QTPP), followed by the identification of the critical elements, including the critical quality attributes (CQAs), associated critical material attributes (CMAs), and critical process parameters (CPPs). All CMAs and CPPs that have an impact on the CQAs of the product are defined and ranked by one of the most important tools of the QbD, which is the risk assessment process [25,26,27]. This step constitutes a platform upon which the planning and designing of the experiment could be built [10].

As there is a lack of real comprehensive work in the literature, the present study aims at the systematic development and optimization of the drying process of a plain mucoadhesive buccal film for the delivery of small or macromolecular drugs, using the solvent casting method by employing QbD principles and the DoE approach. The optimized preparation was formulated with two model drugs, namely, lidocaine hydrochloride (LHCl) and enalapril maleate (EM), to investigate the effect of the presence of API in the film and of possible API–carrier interactions on the properties of the final formulation.

LHCl is a weak base used as a local anesthetic and in the management of arrhythmia and the prevention of acute myocardial infarction. Due to its intensive hepatic first-pass metabolism, there are numerous attempts to deliver it through the buccal route [28,29,30,31].

Enalapril maleate was selected as a second model drug due to its similar physicochemical properties to LHCl. This weak acid prodrug belongs to the angiotensin-converting enzyme inhibitor class of drugs, and it is clinically used in the treatment of hypertension and heart failure. The most common maintenance dose of EM is 20 mg taken orally. It undergoes extensive hepatic first-pass metabolism, but due to its poor absorption (approx. 60% of the administered amount) and conversion (approx. 60% of the absorbed amount), its oral bioavailability is only around 40%. To improve this ratio, there have been several attempts to administer it through the buccal route [32,33,34,35].

## 2. Materials and Methods

### 2.1. Chemicals

High- (average Mw: 1,500,000; batch number: 918VYH) and medium- (average Mw: 1,250,000; batch number: 889EMW) molecular-weight chitosan (CH) (≥90% degree of deacetylation) were purchased from Glentham Life Sciences LTD (Corsham, UK). Glycerol (Gly) (≥99.5%) was supplied by Merck (Darmstadt, Germany), whereas citric acid (CA) (Ph.Eur.8.0; batch number: BX1994654), PEG 400 (Ph.Eur.9.0; Lot No. EC1418626), and lidocaine as lidocaine hydrochloride monohydrate (Ph.Eur.9.0; Lot No. DG8019793; MW: 288.8 g/mol; pKa (amine): 7.9; solubility in water: 50 mg/mL) were purchased from Molar Chemicals Ltd. (Hungary). Enalapril as enalapril maleate (MW: 492.5 g/mol; pKa (acid): 3.0; pKa (amine): 5.5 (in salt form) or 8.0 (in base form); solubility in water: 25 mg/mL) was received as a gift from Richter Gedeon (Budapest, Hungary). Mucin Type II from porcine stomach was purchased from Sigma-Aldrich (Shanghai, China). Distilled water was used as a solvent in the preparation of the solutions.

### 2.2. Preparation of the Buccal Films

In all formulations, the required grade and amount of CH was dispersed in an aqueous CA solution and stirred (Stirrer DLS, VELP Scientifica, Budapest, Hungary) at 800 rpm using a propeller-type mixing rod until a clear viscous solution was obtained. In the drug-loaded film preparations, the required amount of the drug was added to the clear solution of the polymer of the optimized formula and mixed. Then, the assigned amount of the plasticizer, calculated relative to the polymer dry weight, was added to the solution, which was further stirred for about 20 min. A predetermined amount of the solution was poured onto plastic plates (7.5 cm × 7.5 cm), which were then placed in an oven equipped with controllable forced air circulation (Memmert GmbH + Co. KG, Buechenbach, Germany) under predetermined temperature and airflow conditions according to the DoE (Table 1 and Table 2), as discussed in Section 2.4.1 and Section 2.4.2, respectively. The dried films were then carefully removed, sealed in plastic bottles, and stored under ambient conditions until further characterization.

### 2.3. Characterization of the Films

#### 2.3.1. Film Weight and Thickness

The mass of the films (n = 3) was measured on an analytical balance with a measurement capacity of up to 120 g and an accuracy of 0.0001 g (Type PT120, Sartorius, Göttingen, Germany). The thickness of each film was measured using a micrometer screw gauge with a measurement range of 0–25 mm and a resolution of 0.001 mm (Mitutoyo, Kawasaki, Japan) at six different points of each film and the average values and standard deviations were calculated.

#### 2.3.2. Moisture Content Determination (MC)

A halogen moisture analyzer (MAC 50 RADWAG, RADWAG Wagi Elektroniczne, Radom, Poland) was used to determine the MC of a sample of each film formulation. The measurements were based on a thermogravimetric principle, as the mass loss upon drying was recorded and expressed as a percentage of the original mass. The values were determined on the basis of 3 parallel measurements using a 105 °C drying temperature and medium switch-off conditions of the apparatus.

#### 2.3.3. Breaking Hardness (BH)

The BH of the prepared films was measured using a texture analyzer developed at our institute. The equipment has different sample holders and probes based on the type of test. For measuring the film hardness, the sample was fixed with the sample holder at the bottom of the equipment, and a needle-like probe (3 mm diameter) was moved downward at a constant speed (2 mm/s) against the film. The test was finished when the film was pierced and the time of the test and force (range of 0—50 N) required to break the film were recorded. The output was presented in the form of a force–time curve displayed by a computer connected to the equipment. At least 5 parallel measurements were performed on 1 cm^2^ pieces of each casted sample, and the mean values and standard deviations were calculated [36].

#### 2.3.4. Mucoadhesivity (MA)

In vitro MA in terms of detachment force was measured by the same texture analyzer mentioned above with modification in the settings and assembly. The sample was fixed by a double-faced adhesive tape on the bottom surface of a rod-like probe with a diameter of 9 mm. Then, 25 µL of a freshly prepared mucin solution (10% *w*/*w*) was spread on a 35 mm circular disc assembled at the bottom of the equipment. The rod-like probe was moved down towards the bottom disc, and 30 ± 0.1 N force was applied for 30 s to bring the film into intimate contact with the mucin. Following that, the probe was moved upwards, and the force required to detach the film from mucin was presented in the form of a peak in the force–time curve. The detachment force (MA) of the film corresponds to the displayed peak maximum. The means and standard deviations were calculated for each sample from at least five measurements [36].

#### 2.3.5. Drug Content Uniformity Measurement

**Figure 1:**
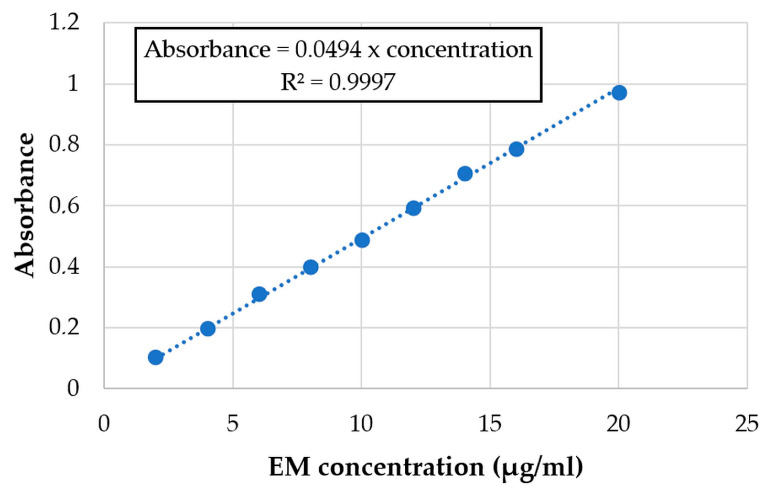
Calibration curve of EM at λ_max_ 209 nm.

**Figure 2:**
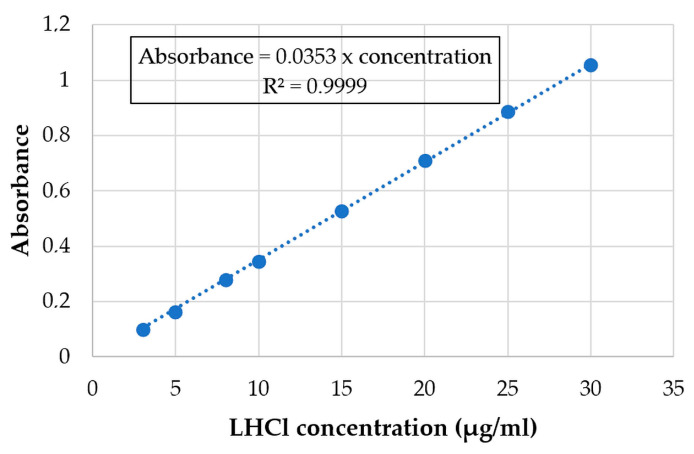
Calibration curve of LHCl at λ_max_ 218 nm.

The wavelength of maximum absorption for EM and LHCl was determined by scanning a dilute aqueous solution of the drugs in the wavelength region of 200–400 nm by a UV-VIS spectrophotometer (Helios Alpha, Unicam, Budapest, Hungary). The λ_max_ was found to be 209 nm and 218 nm for the EM and LHCl, respectively A standard graph was prepared by measuring the absorption of a series of drug solutions of known concentrations. The obtained calibration curves of EM and LHCl are displayed in Figure 1 and Figure 2, respectively.

The content uniformity test was performed to ascertain the uniform distribution of the drug in the polymeric film. Specimens of 1 cm^2^, containing a theoretical amount of 5 mg of EM or LHCl, were cut from each film in 3 different places and dissolved separately in 100 mL of phosphate buffer pH 6.8. The content of the drug in the polymer film was determined spectrophotometrically, and the average drug content of the 3 replicate samples was taken as a final reading.

#### 2.3.6. Evaluation of In Vitro Drug Release

An in vitro drug release study was performed in a USP XXIV 6-station dissolution apparatus type 1 (Erweka DT700 LH, Germany), with 900 mL phosphate buffer pH 6.8 as a dissolution medium, and maintained at 37 ± 0.5 °C at a mixing speed of 100 rpm. Specimens of 1 cm^2^ size drug-loaded films, containing a theoretical amount of 5 mg of EM or LHCI were used in this investigation. Aliquots of 5 mL were withdrawn from each station at a predetermined time, filtered, diluted suitably and then analyzed spectrophotometrically. The cumulative percentage of the released drug was calculated from the calibration curves displayed in Figure 1 and Figure 2, and the absorbance was corrected with the results of the blank film composition. Each determination at each time point was calculated as the average of 3 parallel results, and the error bars of the cumulative curves represent the standard deviation.

#### 2.3.7. In Vitro Permeability Studies

In addition, an in vitro permeability study was carried out using an Enhancer cell through an artificial regenerated cellulose membrane (Visking Dialysis Tubing, pore size diameter Ca. 25 A°, SERVA, Heidelberg, Germany). The size of the measured films was 1 *×* 1 cm. The films were put on the surface of the membrane and placed into the donor compartment, which was 2 mL of phosphate buffer (pH = 6.8). The acceptor compartment was also phosphate buffer (pH = 7.4, 300 mL), simulating the pH of blood. The test was run in an Erweka DT700 dissolution tester at a mixing speed of 100 rpm. Aliquots of 5 mL were taken at 15, 30, 45, 60, 90, 120, 180, 240, 300, 360, 420, and 480 min and analyzed spectrophotometrically. The results are the averages of three replicates.

#### 2.3.8. Interactions among Film Components

To investigate the interactions between the drug and the excipients used in the preparation of the film, Fourier transform infrared spectroscopy (FTIR) was used. The infrared spectra of the drug, excipients, and polymer films were obtained using an FTIR (Avatar 330 FTIR, Thermo Fisher Scientific Inc., Waltham, MA, USA) spectrophotometer coupled with Zn/Se horizontal attenuated total reflectance (HATR) equipment. The films were laid on a clean crystal of the apparatus and scanned for absorbance in the wavelength range of 600 to 4000 cm^−1^. The spectra were obtained from 128 scans with a spectral resolution of 32 cm^−1^ and applying H_2_O and CO_2_ corrections. SpectraGryph (version 1.2.15.; Dr. Friedrich Menges Software, Entwicklung, Obersdorf, Germany) was used in the evaluation of the results.

### 2.4. Choice of Design, Experimental Layout, and Statistical Analysis

The building of the designs, analysis of the data, and drawing of the graphics were executed using the statistical program Statistica, version 13 (TIBCO Software Inc., Palo Alto, CA, USA). The selection of factors and their levels in various designs was based on a detailed risk assessment (Figure 1) based on the evaluation of the literature data, previous experiences of the research group, and preliminary trials [1,2,3,5,16,21,37,38]. The values of the factors’ levels were entered into the software, and subsequently, the number of experiments was determined with the corresponding levels for each factor. Differences at *p* < 0.05 were considered significant by using an analysis of variance (ANOVA) F-test, and the best-fitted models were assigned by the software. The details of the experimental designs are described in the following sections. All experiments were performed in a random order to minimize the effects of uncontrolled factors that could introduce bias in the measurements. Finally, the design space was created in the form of a contour plot by combining the CQAs and critical variables.

#### 2.4.1. Screening Design

As mentioned above, seven factors were depicted that affect the quality of the prepared mucoadhesive films. To investigate their effects, a Plackett–Burman screening design was selected to show the relative importance of each factor concerning the CQAs and to help the identification of some factors, which may be neglected in further investigations to reduce the number of factors and experiments required, thus facilitating the optimization process. In this design, seven factors e.g., the chitosan grade (Mwt), chitosan conc.% (*w*/*v*), plasticizer type, plasticizer conc.% (*w*/*w*), citric acid conc. % (*w*/*v*), amount of solution/plate (g), and drying temp. (°C) were investigated with eight experiments at two levels for each factor. The levels of the independent factors and the arrangements of the experiments are shown in Table 1.

#### 2.4.2. Drying Optimization Design

Based on the results of the screening design, the drying conditions have an impact on the CQAs of the prepared films. The drying was carried out in a ventilated oven (Memmert GmbH + Co. KG, Buechenbach, Germany). In this step, two drying parameters; namely, temperature and airflow, were selected as independent factors, and their effects on the duration of drying and the MA and BH of the formulated films were explored. All other factors levels were fixed based on the results of the Plackett–Burmann design. The applied formula contained 2% (*w*/*v*) medium-molecular-weight CH (Mwt CH), 22.5% (*w*/*w*) plasticizer (in relation to the dry CH weight) and 3.5% (*w*/*v*) CA. Table 2 shows the details of the applied three-level full factorial design of two factors with nine runs.

#### 2.4.3. Film Optimization Design

Based on the output of the screening experiments and after the determination of the optimum drying conditions, three factors were selected for the further optimization of the film composition, namely, the CH concentration, Gly (plasticizer) concentration, and CA concentration. A 2^3^ full-factorial design with a central point was selected to optimize the CQAs of the films, namely, BH and MA. The levels of the independent factors and the layout of the experiments are shown in Table 3.

## 3. Results and Discussion

In the present study, comprehensive research was performed to identify and understand how different CMAs and CPPs of the casting method influence the formulation of buccal films. To achieve the goal, the CQAs of the prepared films were firstly determined. The mechanical properties (breaking hardness (BH)) and mucoadhesivity (MA) were selected as the two most important CQAs of the mucoadhesive buccal films [3,4]. Based on the results of previous experiments, films with a BH above or equal to 10 N were found to be appropriate for comfortable handling by patients, while for MA, 12 N was found as the lowest limit where the carrier could ensure the appropriate residence time for the complete absorption of the drug.

**Figure 3:**
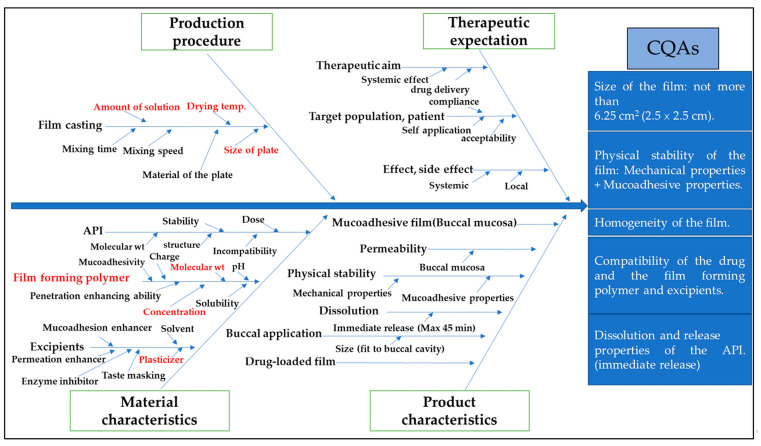
Ishikawa diagram showing the effect–cause relationships among CPPs and CMAs and CQAs of the mucoadhesive buccal formulations.

After that, a thorough risk assessment was done to identify all parameters that could affect the film CQAs. These are highlighted in the form of an Ishikawa diagram (Figure 3). From the listed parameters, seven factors were selected and investigated that were most likely to influence the CQAs of the mucoadhesive buccal films. These included the film-forming polymer grade and its concentration, the type of the plasticizer and its concentration, the drying conditions, and the amount of casted solution used per plate. Based on the aimed therapeutic expectations, chitosan (CH), a physiologically inert and naturally derived, abundant, and inexpensive polymer, was selected as a film-forming polymer by virtue of its biocompatibility, biodegradability, and superior mechanical, penetration-enhancing, enzyme-inhibiting, and mucoadhesive properties [2,3,5,9,11,21,39,40,41,42]. However, due to its chemical nature, this polymer dissolves under slightly acidic conditions. Despite the commonly used HCl or acetic acid, in the present work, citric acid (CA) was used for the acidification of the solution, which is commonly used in the food and pharmaceutical industries [40,42,43] due to its salivary stimulating, plasticizing, penetration-enhancing, antioxidant, and enzyme inhibitory effects [3,17,37,38,40,43]. Thus, its concentration was included as the seventh input variable. The development and optimization of the film formulation were carried out systematically in three steps by employing the DoE approach.

### 3.1. First Step: Screening Experiment

At this step, seven factors were depicted based on the results of the risk assessment to investigate their effects on the CQAs of the mucoadhesive buccal films prepared by the film casting method using the Plackett–Burman screening design.

#### 3.1.1. Film Thickness and Moisture Content

The thickness of the prepared films ranged between 70 µm and 190 µm (Table 4), which is in the ideal range for comfortable application to patients (50–1000 µm) [2,18]. The obtained results show no considerable differences. The only exception was sample F5, which was significantly (*p* < 0.05) thinner than the others. Nevertheless, it could generally be observed that films prepared with medium-molecular-weight chitosan (Mwt CH) were thinner than those prepared with the high-molecular-weight one (Hwt CH). Contrary to what has been reported, which is that the plasticizer imparts increased volume to the film and consequently increases the thickness, a slight but not significant (*p* > 0.05) decrease could be observed in the cases of F1 and F2, although the former had double the amount of PEG400. The same significant (*p* < 0.05) behavior could be seen in the case of Gly when we compared F5 and F6, as well. This could be due to the dominant effect of the polymer over the effect of the plasticizer concentration [10]. The only exceptions were F3 and F4, as the latter was obviously, but not significantly (*p* > 0.05), thinner than the former, although it was prepared with the same concentration of Hwt CH and a double concentration of PEG400. This can be assumed to be an additional factor that affects this characteristic and could be attributed to the higher concentration of CA in the F3 formulation. This assumption is supported by the F5 formula, as it was the thinnest among the prepared films and was prepared by the lower concentration of CA, along with the lower concentration of the Mwt CH. Therefore, this criterion was affected by a group of factors and their interactions.

As the MC affects the physical and mechanical performance of buccal films [44], it was noticed during the preformulation experiments that the films with a MC of less than 2% broke easily upon handling, while the films containing more than 9% MC were very sticky and difficult to handle. Therefore, this range was adopted as optimal to end the drying process and for optimal films.

The MC of the prepared films was between 4.2% and 6.6% (Table 4). As a general trend, the films prepared with the Hwt CH had significantly (*p* < 0.05) higher MC compared to the corresponding formulas prepared with Mwt CH, especially if the polymer was applied in a high concentration. The only exceptions were F1 and F2, where there was no significant change (*p* > 0.05). This could be attributed to the reduced concentrations of both PEG400 and CA. Both compounds had polar groups that bind strongly with water molecules and help to retain water in the film. As the presence of moisture in the film is quite important to prevent films from becoming dry and brittle this could partly explain the exerted plasticizing effect of both compounds [1,18,44]. This explanation could also be supported by another observation when comparing F7 and F8. The latter, formulated with the Hwt CH and higher concentrations of CA and Gly, had a higher MC by approximately 2% compared to the former one.

#### 3.1.2. Breaking Hardness and Mucoadhesivity

As mentioned above, BH and MA were selected as the main CQAs of the formulated films. As can be seen in Table 4, there was a wide variation in the BH, as the maximum value of F4 equals around 8 times that of the minimum of F1. To facilitate the understanding of the effects of the independent factors on this quality, the results were analyzed statistically using Statistica version 13 software, and the following equation was obtained:

BH = 5.331 + 0.164x_1_ + 2.964x_2_ − 0.359x_3_ + 0.271x_4_ − 1.786x_5_ − 0.626x_6_ −1.079x_7_ (1)

**Figure 4:**
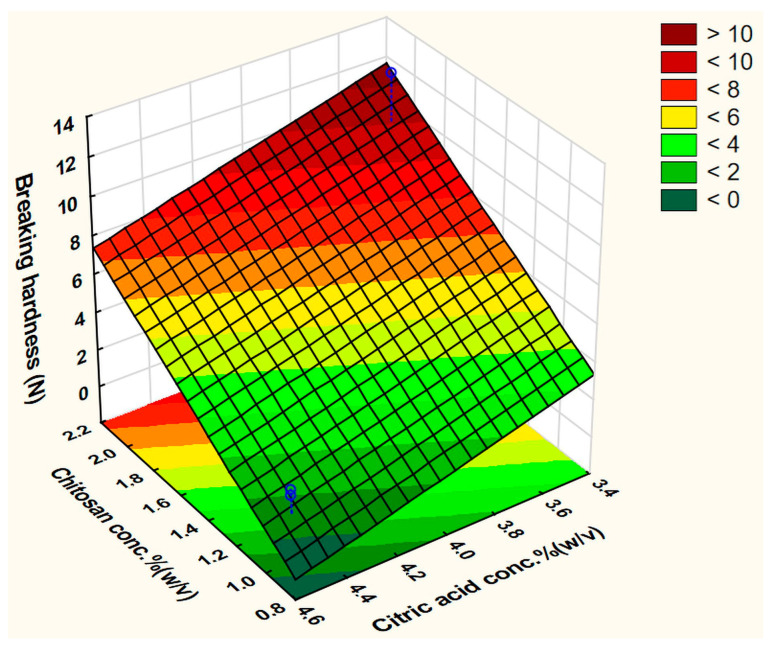
Fitted surface plot of BH in the screening experiment.

This equation represents the full polynomial equation of the no-interaction model and the best-fit equation was obtained by ignoring the least important factor x_1_ (CH grade) with the statistical parameters: R^2^ = 0.99805, adjusted R^2^ = 0.98636, and MS residual = 0.2145125. The CH concentration (x_2_), which has a positive effect, was the only factor that significantly affected the BH. The CA concentration (x_5_) and the drying temperature (x_7_) came as the second and the third most important factors, respectively. In contrast to the other factors, the increased polymer and plasticizer concentrations increased the BH, although the effect of the latter one was negligible. A possible explanation is that CA, which was used to solubilize the polymer, remained in the structure of the film and acted as a plasticizer too. Since it forms a strong ionic bond with CH, it has a stronger influence on the final properties than the other tested plasticizers. It has been reported that PEG and Gly are the best candidates to plasticize CH films [45]. In the present experiment, using Gly resulted in films with higher hardness compared to PEG400. Unexpectedly, using a larger amount of the solution/plate caused a reduction in the response. These findings could be visually elaborated by the construction of a response surface plot as a function of two factors and by setting fixed values to the other parameters (Figure 4). This plot shows an apparent increase in the BH upon utilization of a higher concentration of the film-forming polymer and a reduced concentration of CA.

The MA was measured in terms of the force required to detach the films from the mucin solution and showed less variability compared to the BH (Table 4). All formulations showed relatively high MA values, which agrees with what was reported in the literature [29,36]. This polymer, due to its high content of amino and hydroxyl groups, has a high capacity to absorb water which is highly connected with the ability to adhere to the oral mucosa. In addition, CH is a cationic polymer, with the ability to interact with the negatively charged mucin through electrostatic forces and hydrogen bonding, resulting in strong binding, which is quite important in local drug action and permeation through buccal mucosa [11]. The statistical analysis of the results gave the following polynomial equation of the no-interaction model:

MA = 18.294 + 0.524x_1_ + 1.589x_2_ − 0.134x_3_ + 0.839x_4_ + 0.236x_5_ + 0.201x_6_ + 0.731x_7_ (2)

**Figure 5:**
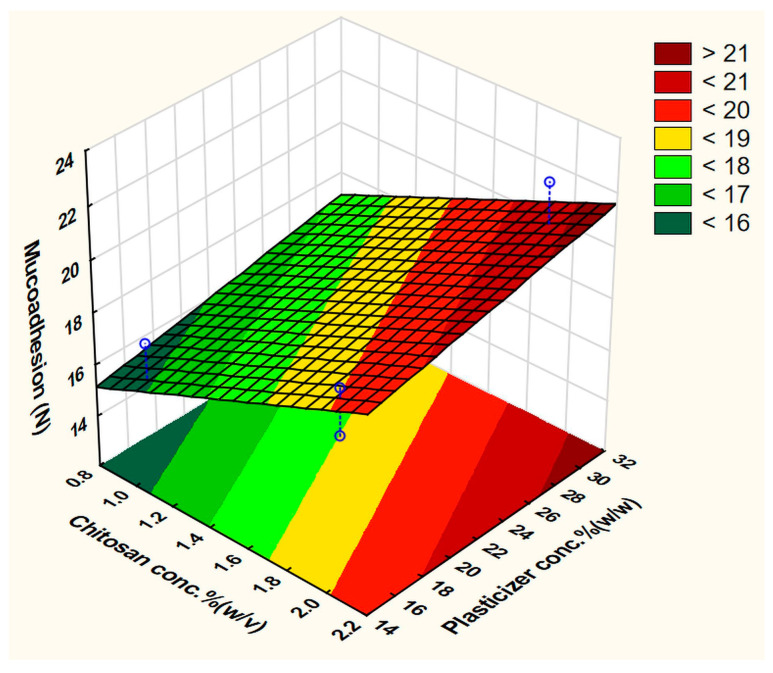
Fitted surface plot of MA in the screening experiment.

The best-fit equation was obtained by ignoring x_3_ (plasticizer type) with the statistical parameters: R^2^ = 0.99569, Adj. R^2^ = 0.96983, and MS residual = 0.1431125. This model did not reveal any significant factor, but the polymer (x_2_) and plasticizer (x_4_) concentrations were the factors with the highest coefficient values. Increasing the CH and plasticizer concentrations improved the MA (Figure 5). In a similar way to the BH, films containing Gly as a plasticizer gave higher MA than those containing PEG400.

Some authors reported a relationship between the MC of the polymer matrix and the detachment force and stated that a higher MC leads to a reduction in this force [11]. This observation could be seen when the pairs of formulations prepared by the same grade of polymer at the same concentration were compared. This is obviously applicable to F2 and F6, and F3 and F7, and less apparent in the case of F1 and F5. The only exception is F4 and F8, and this could be due to the higher concentration of the CA in F8.

As noticed above, the grade of the polymer (x_1_) was found to be low for both responses, and therefore, Mwt CH was selected for the following experiments because more favorable thinner films were obtained, and the casting solution of this grade is easier and faster to prepare. Nevertheless, the effect of the polymer concentration was significant for BH and had the greatest importance for MA, and therefore, it was selected to be included in the optimization step.

Plasticizer as an additive has a fundamental effect on the mechanical behavior of the polymer matrix, and therefore, its concentration needs to be optimized in the formulation. As it can be concluded from both Equations (1) and (2), Gly gave the films higher BH and MA, and hence, it was selected as the plasticizer for the following steps. This selection is supported by the findings of Domján et al., who reported that Gly binded energy H-bonds with glucosamine units of CH three times more compared to PEG400, which reflects stronger second-order bonds between CH and Gly molecules. In other words, Gly acts much more as an internal plasticizer for CH films, while PEG400 rather acts as an external plasticizer [45].

The CA concentration, along with the plasticizer concentration, showed great importance in both responses, especially for CA, which showed inconsistent effects on the CQAs, and thus, they were selected to be investigated further in the following experiment. On the other hand, the amount of the solution/plate did not show any significance. It had a positive effect on the detachment force, while it harmed the BH. Thus, the amount was fixed in the following preparations at the 0 level of 20.5 g (0.364 g/cm^2^). The drying temperature was the third most important factor in the case of the film hardness, and consequently, the conditions of the drying process, which is a time-consuming step, were decided to be optimized in a separate design.

### 3.2. Second Step: Drying Optimization Experiment

The effects of temperature and airflow on the drying process were investigated at three levels. The output of the nine runs of the full factorial design investigating three responses is shown in Table 5, while the weights of films, their thicknesses, and MCs are displayed in Table 6.

The main target of this experiment was to prepare films of the acceptable CQAs within a short time. When the lowest level of ventilation and temperature used in Run 1 increased to the highest level in Run 9, the drying time was reduced to around 6 h. However, the fast drying negatively affected the BH. The statistical analysis showed that the two-way interaction model described well the effects of the drying factors on the three selected criteria. In the case of the DT, the following polynomial equation was obtained:

DT = 416.667 − 77.5x_1_ + 6.25x_1_^2^ − 95x_2_ − 5x_2_^2^ + 3.75x_1_x_2_ − 9.375x_1_x_2_^2^ + 9.375x_1_^2^x_2_ + 17.813x_1_^2^x_2_^2^ (3)

**Figure 6:**
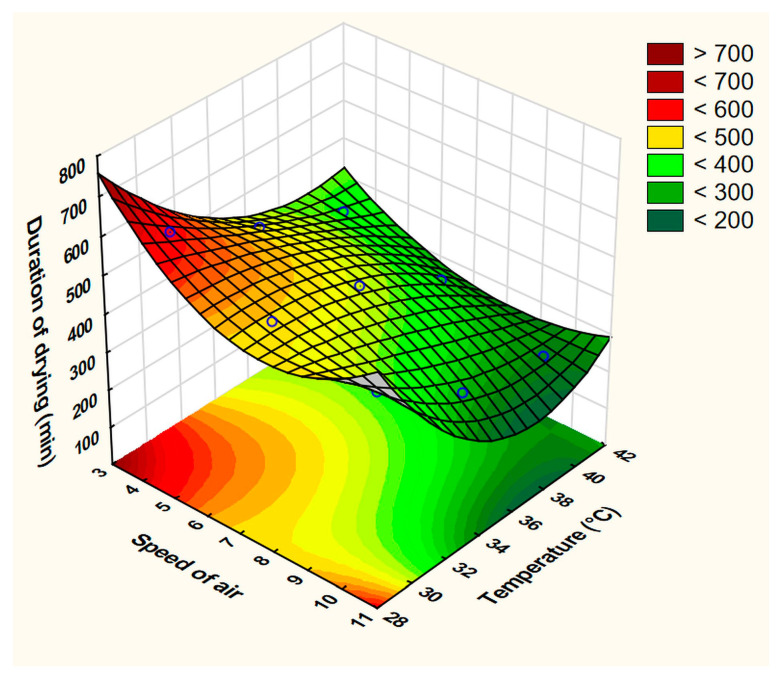
Fitted surface plot of the dependent variable duration of drying (min).

The best-fit equation was obtained by ignoring the linear interaction x_1_x_2_ with the statistical parameters: R^2^ = 0.9994, Adj. R^2^ = 0.99521, and MS residual = 56.25. Both the ventilation level (x_1_) and applied temperature (x_2_) significantly affected the time required to dry the films. This can be seen clearly from the surface plot (Figure 6), as moving above the average values of the speed of air and temperature led to a drying time of less than 10 h.

The polynomial equation of the BH response is as follows:

BH = 19.997 − 1.827x_1_ + 5.57x_1_^2^ − 6.05x_2_ + 2.15x_2_^2^ − 2.155x_1_x_2_ + 0.823x_1_x_2_^2^ − 3.098x_1_^2^x_2_ + 2.464x_1_^2^x_2_^2^ (4)

**Figure 7:**
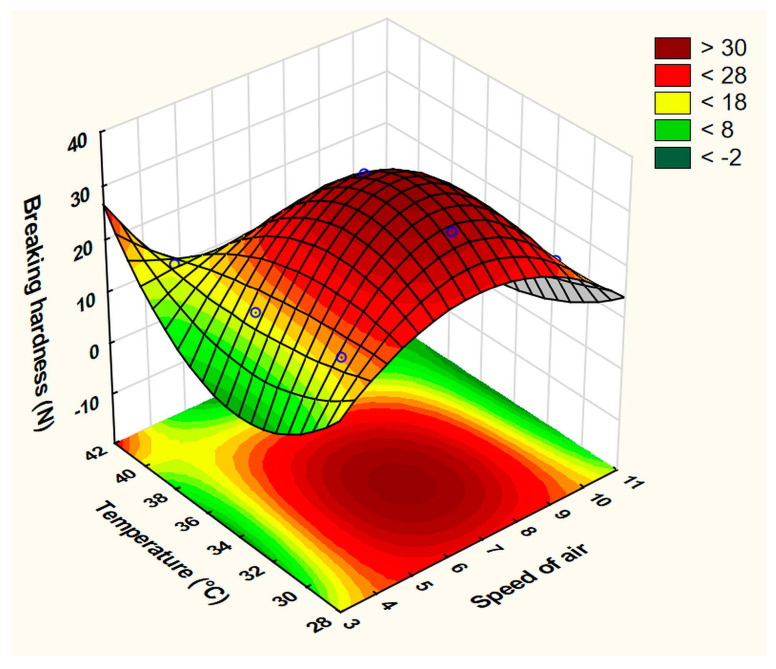
Fitted surface plot of BH in the drying process optimization experiment.

The best-fit equation was obtained by ignoring the interaction factor x_1_x_2_^2^ with the statistical parameters of R^2^ = 0.99437, Adj. R^2^ = 0.95499, and MS residual = 3.608. Although this equation did not reveal any significant factor, the effect of the drying temperature was the greatest and can be visualized by the surface plot (Figure 7).

**Figure 8:**
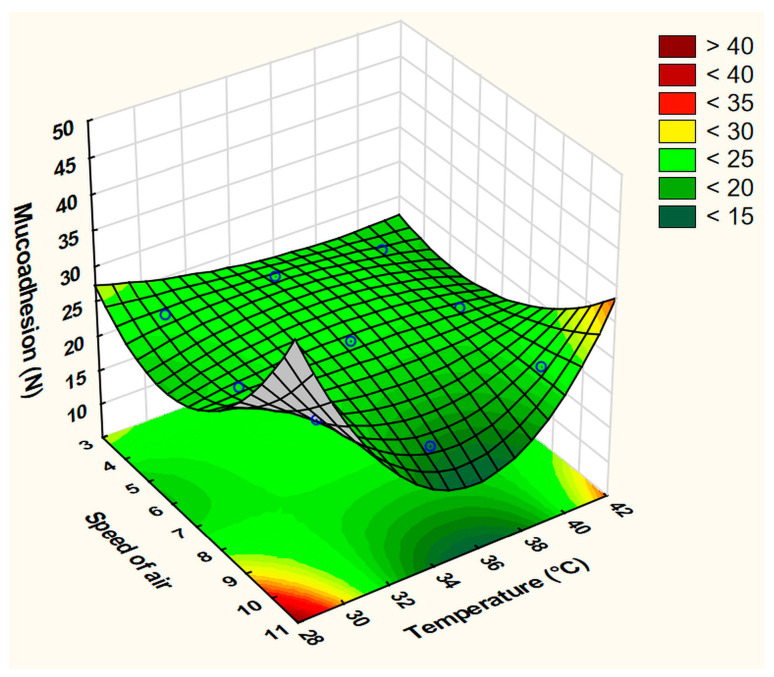
Fitted surface plot of MA in the drying process optimization experiment.

The variability in the MA among the nine runs was found to be smaller compared to the previous responses (Table 5, Figure 8). This response can be described by the following equation:

MA = 22.911 − 0.178x_1_ − 0.126x_1_^2^ − 1.16x_2_ − 0.998x_2_^2^ − 0.725x_1_x_2_ − 2.075x_1_x_2_^2^ + 0.743x_1_^2^x_2_ + 1.023x_1_^2^x_2_^2^ (5)

The best-fit equation was obtained after omitting the quadratic factor of the ventilation level (x_1_^2^), which had high R^2^ = 0.99755 and Adj. R^2^ = 0.98044 values and a low MS residual of 0.1267. The interaction factor (x_1_x_2_^2^) significantly affected the quality of the prepared films.

As shown in the previous section, there was a relationship between the MC and the CQAs of the film. In all preparations, the MC was within the adopted optimum range (Table 6). It was noticed that as the upper limit was approached, for example, in Runs 1 and 5, the handling of the films became more difficult. All films were relatively thin ranging from 156 µm to 185 µm. However, it was difficult to establish a relationship between the thickness and the MC and between the thickness and the applied temperature and airflow speed. The weights of the produced films were approximately similar with a maximum RSD of around 5%.

**Figure 9:**
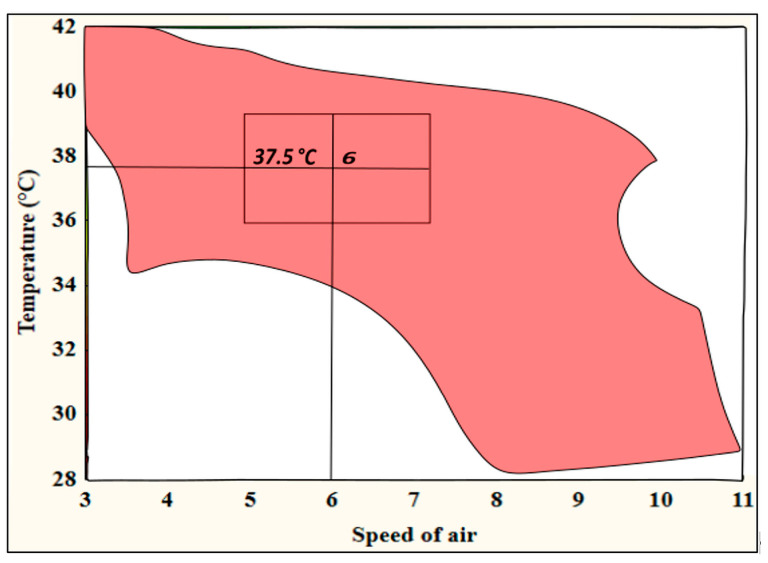
Contour plot of the optimized film drying process design space. The rectangle within the design space represents the control space.

The optimized conditions of the drying process could be obtained by building up a design space that represents all possible combinations and interactions of the process parameters that result in responses within the required range, which is recommended by the regulatory bodies [25]. The target for the three responses was set to DT ≤ 8 h, BH ≥ 10 N and MA ≥ 12 N (colored zone, Figure 9).

A control space was determined within the design space (rectangle) to impose more control on the quality of the produced films. In the center of the control space, a point was depicted and selected to be the suitable values of the temperature and airflow speed (37.5 °C, 6) that would be employed in future experiments. Within the design space, lower temperatures could be used when the ventilation level is increased above 8 (lower right corner). However, higher levels of airflow speed might result in films of less uniform thickness, and consequently, medium values within the selected control space are preferred.

### 3.3. Third Step: Film Optimization Experiment

Optimization of the composition was the last step in this sequence for the development of buccal film formulation. Based on the output of the screening study and after the determination of the optimum drying conditions, three factors were selected for the optimization step, namely, the CH concentration, Gly (plasticizer) concentration, and CA concentration. A set of nine experiments was performed, constituting a 2^3^ full-factorial design with a central point to fine-tune the levels of the three factors that produced films with the required CQAs.

#### 3.3.1. Weight, Moisture Content, and Thickness of Films

The variation in the weight of films was mainly attributed to the variability in the weights of the three materials in different formulations set by the design (Table 7). As can be observed, the major contributors to weight were CA and CH, respectively. However, the relationship between the weight and the thickness was not obvious. For example, Formulas 5 and 9 had almost the same weight, although there was a clear difference in their thickness. On the other hand, Runs 4 and 5 had almost the same thickness, but there was an apparent difference in their film weight. The highest values of thickness appeared in Runs 6 and 8, in which higher levels of the polymer and CA were used, indicating that the polymer and CA concentrations were the most important factors controlling the thickness.

Generally, moving towards the higher CH concentration caused an apparent increase in the film’s thickness, which is reminiscent of the previous reports’ findings [10,20]. The positive contribution of the excipients started to appear at higher levels of CH (Runs 5–8). For instance, when their concentrations were simultaneously increased from lower levels (Run 5) to a higher level (Run 8), the average value of the film thickness increased by 83 µm. This could be justified by the disruption and restructuring of the polymer chains caused by these excipients causing an increase in the occupied volume and, consequently, imparting thickness to the prepared films [10,21].

Table 7 shows that there were differences in the MC among the runs in the range of 1.54%. There was also no clear relationship between the MC and thickness. For instance, although Runs 3 and 5 had similar film thickness, Run 3 had the highest retained moisture, while Run 5 had the lowest MC.

#### 3.3.2. Breaking Hardness and Mucoadhesivity

All formulations with lower concentrations of CH (Runs 1–4) were stickier and relatively difficult to cast and handle, especially when the CA was at a higher level. It was generally observed that tougher films were obtained at the higher CH concentrations (Table 7). The statistical analysis showed that the three-way interaction model was the best-fit equation and describes well the effects of the three factors on the BH:

BH = 11.048 − 3.620x_1_ − 2.915x_2_ + 7.083x_3_ + 0.908x_1_x_2_ − 2.450 x_1_x_3_ − 3.065x_2_x_3_ + 0.958 x_1_x_2_x_3_ (6)

All three main effects (x_1,_ x_2_, and x_3_) significantly affected the response and some interaction factors, namely, x_1_x_3_ and x_2_x_3_. The values of the R^2^, Adj. R^2^, and MS residual were 0.9998, 0.99838, and 0.1440056, respectively. Since the curvature in the model was not significant, the design and the three-way model were satisfactory in describing the behaviors and effects of these factors on the response within the experimental domain without the need for higher-level or more sophisticated designs.

The statistical analysis supported the observation that the concentration of the polymer significantly affected the BH along with its interaction with the CA (x_1_x_3_) and Gly (x_2_x_3_). As shown in Table 7, the simultaneous use of high levels of CA and Gly led to a dramatic reduction in the BH independently from the applied CH concentration. This observation can be understood by the equation, as the main factors of both excipients (x_1_ and x_2_) were inversely proportional to this response. It is noteworthy to highlight that in addition to its acidification function, the residual free CA can also act as a plasticizer through the formation of hydrogen bonds between the carboxylic groups on CA and the polar groups of the polymer [46]. Thus, both excipients act synergistically to improve the mobility of the polymer chains, leading to increased flexibility, and hence, apparent reduction of the BH. This agrees with the findings of Calvo et al. that the amount of plasticizer was among the factors with the greatest effect on the film BH [21]. Furthermore, Visser et. al. showed that a high percentage of Gly resulted in stickier films with decreased tensile strength, and a similar effect was stated by Uranga et al. after the addition of CA [20,38], thereby corroborating our findings and interpretation.

Similarly, the MA was described by the three-way interaction model:

MA (N) = 13.719 + 0.134x_1_ + 0.649x_2_ + 0.549x_3_ − 0.819x_1_x_2_ + 0.421x_1_x_3_ + 0.076x_2_x_3_ − 0.866x_1_x_2_x_3_ (7)

The best-fit of the polynomial equation was obtained by ignoring the CA concentration (x_1_) and the interaction factor (x_2_x_3_), and the statistical parameters were: R^2^ = 0.9752, Adj. R^2^ = 0.93385, and MS residual = 0.1573755. Again, as can be observed in Table 7, the effect of the CH concentration was revealed to be significant, along with the Gly concentration and interaction factors x_1_x_2_ and x_1_x_2_x_3_, while the curvature was not significant. The effect of the excipient on this CQA appeared to be more complicated, as the main factors were directly proportional to the MA, while their linear interaction factor was inversely proportional.

**Figure 10:**
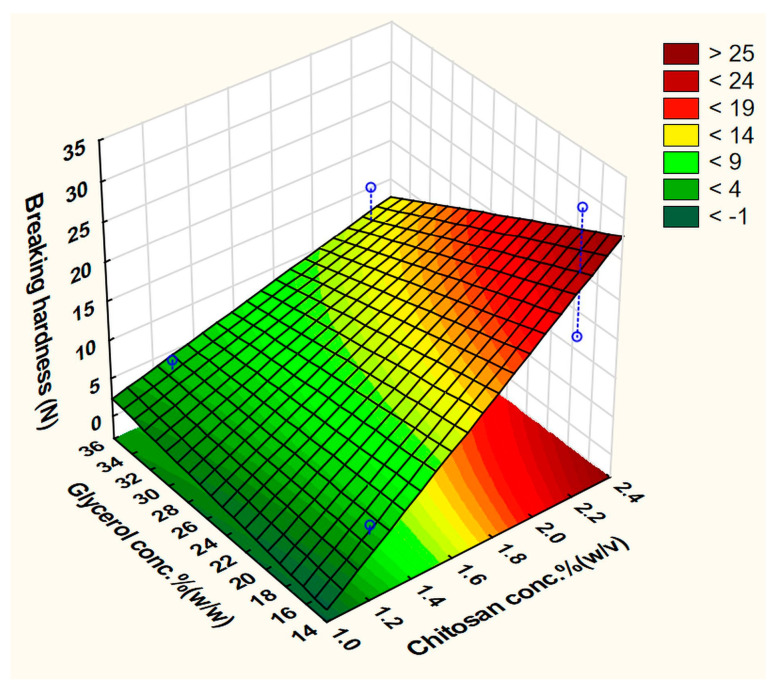
Fitted surface plot of the dependent variable BH (N) in the buccal film optimization step.

**Figure 11:**
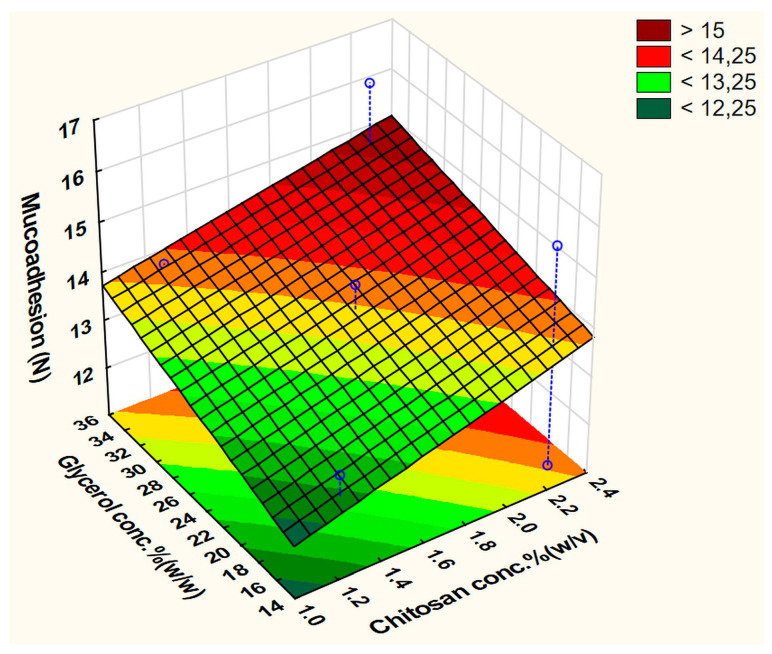
Fitted surface plot of the dependent variable MA (N) in the buccal film optimization step.

The surface plots (Figure 10 and Figure 11) visually show the apparent positive effects of the increasing CH concentration on the BH and MA of the prepared films.

However, the effect of the plasticizer at the fixed average value (4%) of the other excipient was controversial, as it inversely affected the BH, while a less important positive effect was exerted on the MA response. These effects agree with previous reports, as the polar groups in Gly help in potentiating the water uptake capability and increasing the flexibility of CH chains, and thus, enhance its interactions with buccal mucosa, and more specifically, mucin. Gly, which behaves more like an internal plasticizer, binds strongly with the polymer chains, leading to increased elasticity and reduced brittleness [4,10,11,12].

#### 3.3.3. Design Space of the Optimized Film Formulation

**Figure 12:**
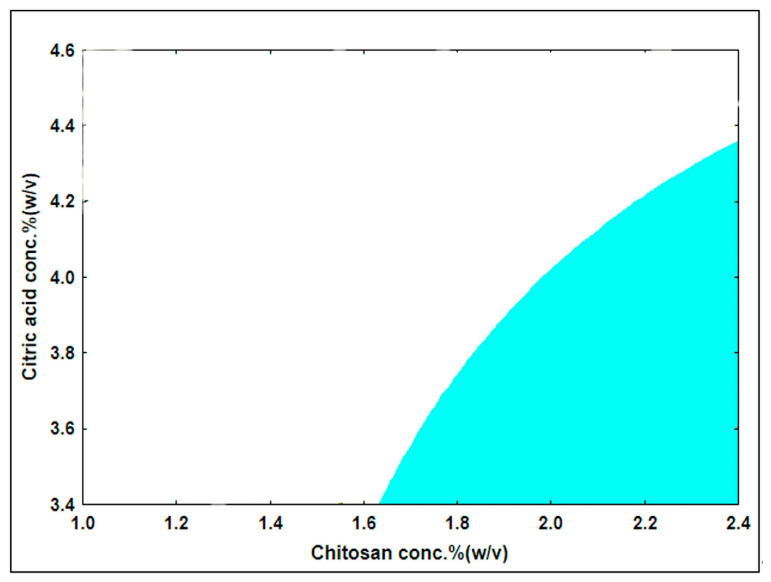
Design space of the CH-based mucoadhesive buccal film (Gly concentration = 35%).

A design space describing the relationship between the input variables and CQAs of the buccal film was built at the end of this step, with the same acceptance ranges of BH ≥ 10N and MA ≥ 12N as in the previous steps. Figure 12 illustrates the design space (colored zone) at the higher level of Gly (35%). From this design space, any combination of the input variables could be selected and employed in future preparations. A combination with the values of CA = 3.7%, Gly = 35%, and CH = 2.1% was selected as an optimized formula.

### 3.4. Drug-Loaded Films

After the optimization step, the optimized formula was loaded with model drugs EM and LHCl to investigate the effect of the presence of API and possible drug–carrier interactions on the properties of the plain mucoadhesive films. An amount of EM was added to the film solution such that the 2 × 2 cm^2^ size of the film contained 20 mg of the API, which is equivalent to the marketed 20 mg EM tablet. The same amount was used to formulate the LHCl films. Table 8 shows the results of the physical properties, MA, and BH of the optimized drug-free and drug-loaded films. The results of the drug-free film fulfil the predetermined CQAs. The EM film showed improved MA and BH compared to the drug-free film. However, the LHCl film showed a dramatic increase in the BH—almost double that of the drug-free film, while its MA dropped to about half of the predetermined value in the CQAs. This reduction in the mucoadhesive properties of the LHCl films is in contrast to the findings of Dahl et. al., which could be attributed to the different polymeric materials they used [34].

It is also clearly visible from the data displayed in Table 8 that the loading of the drug caused an apparent increase in the film thickness. The EM and LHCl films had similar weight and moisture content, but the latter resulted in relatively thicker films.

#### 3.4.1. API-Carrier Interactions

**Figure 13:**
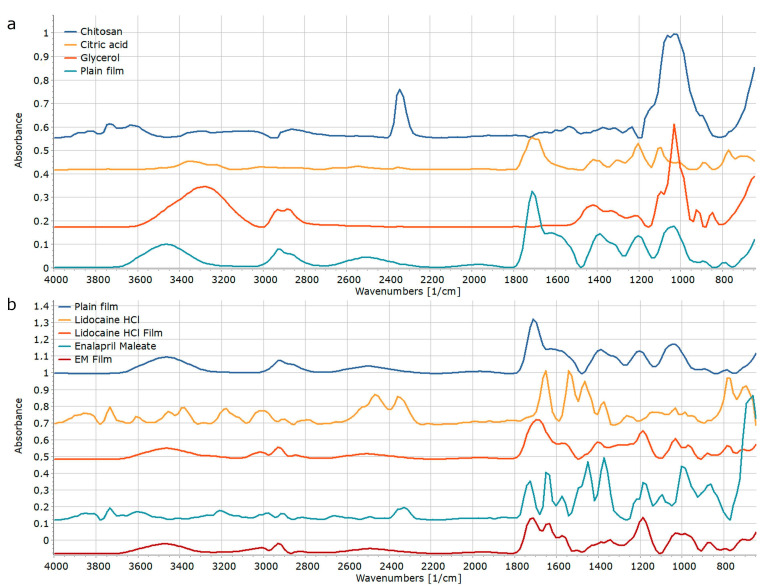
FT-IR spectra of raw materials and formulation in case of the plain (**a**) and drug-containing (**b**) films.

The differences in the properties of API-containing films may be well explained with the differences in the interaction between the API and carrier materials. Figure 13 displays the FT-IR spectra of the raw materials and film formulations. Each displayed spectrum was obtained by the averaging of three or six individual measurements in the case of raw materials and films, respectively. The similarities among the individual spectra indicate a uniform distribution of the drug within the buccal film.

The broad peaks between 1900–2100 and 2300–2700 cm^−1^ (Figure 13a) indicate partial ionization of the CH as result of an interaction with citric acid, as it is indicated by the right shift of the C=O stretching from 1677 cm^−1^ to 1620 cm^−1^, but the unchanged position of the second C=O stretching peak at 1710 cm^−1^ indicates that there were still free acidic groups in the plain film [47,48].

The presence of free acidic groups plays considerable role in the formation API–carrier interactions, as is clearly visible in Figure 13b. The right shift of the C=O stretching of CA from 1710 cm^−1^ to 1688 cm^−1^, and the left shift of the amide II (O-C-N) peak of LHCl from 1536 cm^−1^ to 1567 cm^−1^ indicates weak electrostatic interactions between the API and the carrier [49]. In contrast, all characteristic signals at 1726, 1710, 1645, and 1577 cm^−1^ (C=O stretching of EM’s COOH, CA, EM’s COOEt, and EM’s N-H bend, respectively) are visible in unchanged position in the spectrum of EM containing film, which indicates no API–carrier interaction in this system [50]. The presence or lack of interactions may answer the differences in the MA of the various systems. The slight increase in the MA of EM-containing films may be explained by the presence of free acidic groups of the API, which may also interact with certain functional groups of mucin molecules. In contrast, the LHCl binds the free acidic groups of CA, and thus, decreases the interaction capacity of the final formulation with mucin molecules.

#### 3.4.2. Uniformity of Drug Content

The λ_max_ was found to 209 nm and 218 nm for EM and LHCl, respectively. The content% of the drug calculated from three specimens taken from different places was 97.28 ± 3.17% and 97.95 ± 2.53% (theoretical amount = 5 mg/1 cm^2^) for the EM and LHCl, respectively. These results, like the FT-IR spectra, indicate the uniform distribution of APIs in the mucoadhesive film. Apparently, a higher percentage of the APIs was loaded into the polymer film compared to in a previous report that formulated an EM film for buccal delivery [32].

#### 3.4.3. In Vitro Drug Release

**Figure 14:**
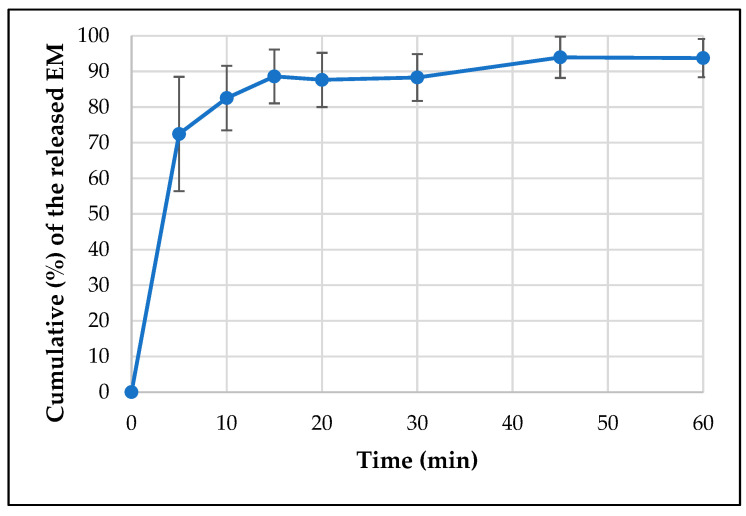
Dissolution curve of the EM-loaded film.

**Figure 15:**
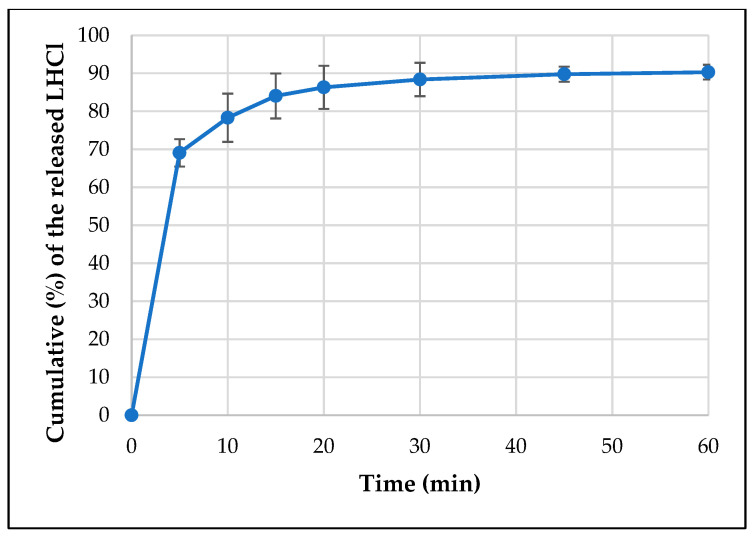
Dissolution curve of the LHCl-loaded film.

An in vitro release study for the API-loaded films was performed in phosphate buffer pH 6.8 using a type I dissolution tester. The cumulative% of the released API is shown in Figure 14 and Figure 15. Around 85% of the loaded API was released in just 10 min and the whole loaded amount was released in about 45 min in case of both APIs. This represents an immediate-release buccal mucoadhesive film, which agrees with the predetermined specification in the Ishikawa diagram (Figure 1).

#### 3.4.4. In Vitro Permeability Test

**Figure 16:**
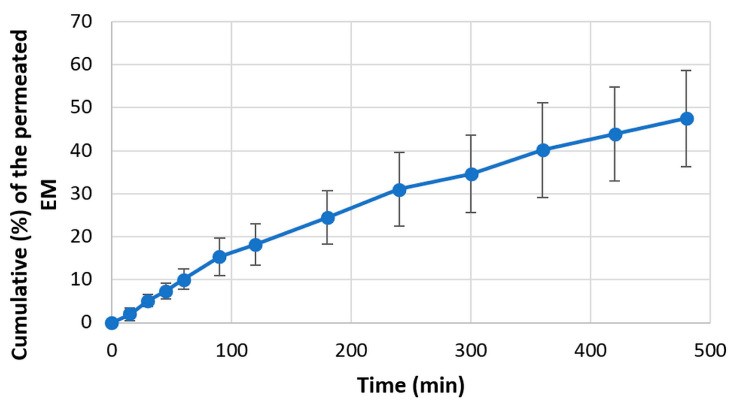
Permeability curve of EM.

**Figure 17:**
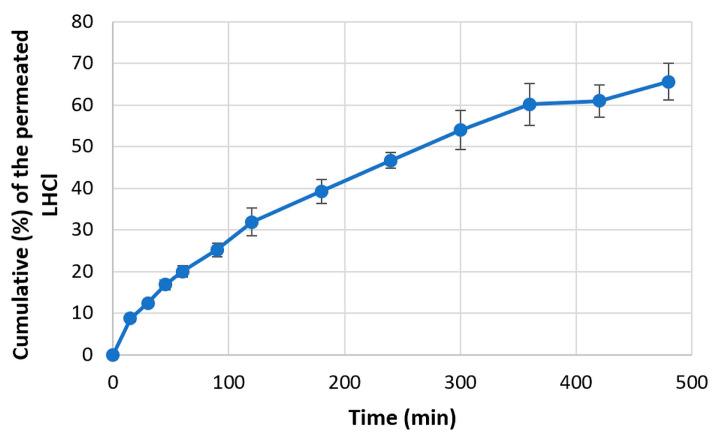
Permeability curve of LHCl.

The results of in vitro permeability of EM and LHCl through an artificial regenerated cellulose membrane are displayed in Figure 16 and Figure 17, respectively. It is clearly visible that despite the almost identical dissolution results, the permeated amount of acidic EM was considerably smaller in comparison to the LHCl, and higher deviations of the results may be observed. This might indicate an interaction between the EM and the applied membrane, hindering the diffusion of the API. This might be a considerable drawback for such APIs since the permeation rate was found to be slightly slower than would be ideal for delivery. However, it should be noted that the permeation-enhancing effect of the applied carrier system might not be utilized on the applied artificial membrane.

## 4. Conclusions

To the best of our knowledge, this is the first comprehensive work that studied the effects of seven independent variables in the development and optimization of mucoadhesive buccal films by applying the QbD principles and focusing on the drying conditions. QbD, along with the DoE approach, appears to be a highly efficient systematic way to rationally design mucoadhesive buccal films. The Mwt CH was found to be preferable over the Hwt one by easier and faster preparation of the casting solution, as well as thinner produced films. The concentration of CH was the most important factor affecting the CQAs. Gly was superior to PEG400 in producing films with better BH and MA. The suitable amount of the casting solution/plate was selected to be 0.364 g/cm^2^. The complex relation between the temperature and airflow on the CQAs of buccal films was also evaluated for the first time in this article. The drying conditions were determined and optimized in the form of a contour plot representing the design space of the DT (≤8 h), BH (≥10 N), and MA (≥12 N). The design space of the optimized film formulation was successfully achieved. The thickness of all films, which was affected predominantly by the concentration of the polymer, was within the recommended range, and an MC between 2% and 9% was found to be suitable for the prepared films in terms of their handling and physical stability. The optimized plain carrier system seems to be suitable to deliver 20 mg of API, fulfilling the predetermined CQA, as it was observed in case of the EM-loaded compositions. Nevertheless, the potential API–carrier interactions should be taken into account, especially in case of MA, since this property dropped out of the specification in the case of the LHCl-containing composition. However, both drug-loaded films showed high drug loading, uniform drug distribution, immediate pattern of drug release, and slightly slow but appropriate permeability.

## Figures and Tables

**Table 1:** Plackett–Burman screening design containing seven factors at two levels and eight runs.

Formula No.	Chitosan Grade (Average Mwt) (x_1_)	Chitosan Conc.% (*w*/*v*) (x_2_)	Plasticizer Type * (x_3_)	Plasticizer Conc.% (*w*/*w*) (x_4_)	Citric Acid Conc. % (*w*/*v*) (x_5_)	Amount of Solution/Plate (g) (x_6_)	Drying Temp. (°C) (x_7_)
F1	650	1	1	30	4.5	25	30
F2	1500	1	1	15	3.5	25	40
F3	650	2	1	15	4.5	16	40
F4	1500	2	1	30	3.5	16	30
F5	650	1	2	30	3.5	16	40
F6	1500	1	2	15	4.5	16	30
F7	650	2	2	15	3.5	25	30
F8	1500	2	2	30	4.5	25	40

* 1 denotes Gly and 2 denotes PEG 400.

**Table 2:** Full factorial design of the drying conditions in the optimization experiment.

Run	Airflow * (x_1_)	Temperature (°C) (x_2_)
1	4	30
2	4	35
3	4	40
4	7	30
5	7	35
6	7	40
7	10	30
8	10	35
9	10	40

* Set values equal to 40, 70, and 100% fan capacity, respectively.

**Table 3:** Two-level full factorial design with a central point of the film optimization step.

Run	Citric Acid Conc. % (*w*/*v*) (x_1_)	Plasticizer Conc. % (*w*/*w*) (x_2_)	Chitosan Conc. % (*w*/*v*) (x_3_)
1	3.5	15	1.25
2	4.5	15	1.25
3	3.5	35	1.25
4	4.5	35	1.25
5	3.5	15	2.25
6	4.5	15	2.25
7	3.5	35	2.25
8	4.5	35	2.25
9	4.0	25	1.75

**Table 4:** The results of the Plackett–Burman screening design of breaking hardness (BH), mucoadhesivity (MA), thickness, and moisture content (MC).

Formula No.	Breaking Hardness (N)	Mucoadhesivity (N)	Thickness (µm)	Moisture Content (%)
F1	1.5 ± 0.18	16.86 ± 0.48	128 ± 15	5.70 ± 0.49
F2	2.7 ± 0.35	17.22 ± 1.57	138 ± 21	5.13 ± 1.43
F3	5.98 ± 0.97	19.42 ± 2.90	132 ± 26	4.15 ± 0.02
F4	12.58 ± 1.02	20.21 ± 1.09	117 ± 19	5.30 ± 0.83
F5	3.45 ± 0.41	17.18 ± 1.50	70 ± 18	4.23 ± 1.57
F6	1.82 ± 0.25	15.56 ± 1.43	115 ± 24	5.39 ± 0.24
F7	9.74 ± 1.13	17.62 ± 1.13	160 ± 19	4.69 ± 0.04
F8	4.88 ± 0.33	22.28 ± 2.18	190 ± 18	6.57 ± 0.58

**Table 5:** Results of the full factorial design of the drying optimization experiments at three levels and in nine experiments through the evaluation of the parameters: drying time (DT), breaking hardness (BH), and mucoadhesivity (MA).

Run	Drying Time (DT) (min)	Breaking Hardness (N)	Mucoadhesivity (N)
1	600	20.15 ± 3.58	23.84 ± 2.21
2	480	17.69 ± 1.49	23.70 ± 0.86
3	390	16.49 ± 1.72	21.98 ± 1.65
4	495	33.98 ± 2.66	22.67 ± 1.91
5	450	34.67 ± 3.65	23.23 ± 2.64
6	330	13.62 ± 1.12	22.33 ± 2.20
7	450	19.71 ± 2.01	27.70 ± 1.57
8	300	16.23 ± 2.84	17.81 ± 1.23
9	255	7.43 ± 0.96	22.94 ± 1.89

**Table 6:** Physical characteristics of film weight, moisture content, and thickness for the films in the drying optimization experiment.

Run	Film Weight (g)	Moisture Content (%)	Thickness (µm)
1	1.30 ± 0.02	8.23 ± 0.48	185 ± 35
2	1.31 ± 0.00	5.10 ± 1.39	158 ± 15
3	1.31 ± 0.01	5.64 ± 0.28	163 ± 26
4	1.32 ± 0.01	5.31 ± 0.40	157 ± 24
5	1.28 ± 0.00	7.99 ± 0.92	169 ± 22
6	1.29 ± 0.01	5.09 ± 0.39	156 ± 22
7	1.33 ± 0.01	5.43 ± 0.85	176 ± 12
8	1.32 ± 0.00	5.72 ± 0.61	161 ± 30
9	1.34 ± 0.01	7.17 ± 0.35	157 ± 37

**Table 7:** Results of the nine runs of the full-factorial design of the film optimization experiment at two levels and central point parameters to evaluate: breaking hardness (BH), mucoadhesivity (MA), moisture content (MC), thickness, and film weight.

Run	Breaking Hardness (N)	Mucoadhesivity (N)	Moisture Content (%)	Thickness (µm)	Film wt. (g)
1	4.98 ± 1.00	12.87 ± 1.89	5.27 ± 0.23	150 ± 19	1.11 ± 0.01
2	2.74 ± 0.47	12.2 ± 1.71	5.33 ± 0.23	147 ± 19	1.20 ± 0.01
3	5.38 ± 1.20	13.92 ± 1.67	6.08 ± 1.60	130 ± 12	1.05 ± 0.02
4	2.94 ± 0.25	13.44 ± 2.19	5.75 ± 0.49	132 ± 19	1.22 ± 0.01
5	32.09 ± 4.24	11.24 ± 1.15	4.54 ± 0.55	133 ± 19	1.37 ± 0.01
6	16.22 ± 1.32	15.72 ± 0.77	5.41 ± 0.17	191 ± 19	1.59 ± 0.01
7	16.4 ± 1.96	16.06 ± 1.11	5.09 ± 1.61	179 ± 20	1.43 ± 0.01
8	7.99 ± 0.9	13.8 ± 2.34	5.29 ± 0.98	216 ± 18	1.64 ± 0.02
9	10.69 ± 1.97	14.22 ± 2.64	5.82 ± 1.30	174 ± 28	1.36 ± 0.00

**Table 8:** Physical and mechanical properties of the optimized formula (CA = 3.7%, Gly = 35%, and CH = 2.1%) for the plain and drug-loaded films.

Film	Breaking Hardness (N)	Mucoadhesivity (N)	Moisture Content (%)	Thickness (µm)	Film wt. (g)
Plain film	13.07 ± 2.56	16.15 ± 0.66	7.38 ± 0.17	179 ± 23	1.36 ± 0.01
EM film	19.15 ± 3.27	18.80 ± 3.47	5.12 ± 1.85	222 ± 33	1.66 ± 0.01
LHCl film	26.72 ± 3.80	6.27 ± 1.04	4.93 ± 0.61	246 ± 29	1.69 ± 0.01

## Data Availability

The data can be shared up on request.
